# Effect of Hypoxia on Branching Characteristics and Cell Subpopulations during Kidney Organ Culture

**DOI:** 10.3390/bioengineering9120801

**Published:** 2022-12-14

**Authors:** Morgan Hamon, Hsiao-Min Cheng, Ming Johnson, Norimoto Yanagawa, Peter V. Hauser

**Affiliations:** 1 Medical and Research Services, Greater Los Angeles Veterans Affairs Healthcare System at Sepulveda, North Hills, CA 91344, USA; 2 Department of Medicine, David Geffen School of Medicine, University of California Los Angeles, Los Angeles, CA 90095, USA

**Keywords:** tissue engineering, developmental biology, kidney, physiology

## Abstract

During early developmental stages, embryonic kidneys are not fully vascularized and are potentially exposed to hypoxic conditions, which is known to influence cell proliferation and survival, ureteric bud branching, and vascularization of the developing kidney. To optimize the culture conditions of in vitro cultured kidneys and gain further insight into the effect of hypoxia on kidney development, we exposed mouse embryonic kidneys isolated at E11.5, E12.5, and E13.5 to hypoxic and normal culture conditions and compared ureteric bud branching patterns, the growth of the progenitor subpopulation hoxb7+, and the expression patterns of progenitor and differentiation markers. Branching patterns were quantified using whole organ confocal imaging and gradient-vector-based analysis. In our model, hypoxia causes an earlier expression of UB tip cell markers, and a delay in stalk cell marker gene expression. The metanephric mesenchyme (MM) exhibited a later expression of differentiation marker FGF8, marking a delay in nephron formation. Hypoxia further delayed the expression of stroma cell progenitor markers, a delay in cortical differentiation markers, as well as an earlier expression of medullary and ureteral differentiation markers. We conclude that standard conditions do not apply universally and that tissue engineering strategies need to optimize suitable culture conditions for each application. We also conclude that adapting culture conditions to specific aspects of organ development in tissue engineering can help to improve individual stages of tissue generation.

## 1. Introduction

Oxygen is an essential component in most metabolic and cellular processes. Low oxygen, or physiological hypoxia, often has a pathological effect on exposed tissue or cells. Normal oxygen (O_2_) concentration in the adult artery is 10–13% (partial pressure PO_2_ of 75–100 mm Hg), while physiologic hypoxia is defined as an O_2_ concentration of 1–5% (8–38 mm Hg PO_2_) [1]. Oxygen also has an essential role in embryonic development. During the early stages of development, the embryo is exposed to a physiologic hypoxic environment [2]. Physiologic hypoxia might be essential for development since in vitro studies have shown that hypoxia has a proliferative effect on embryonic stem cells (ESC) [3] and a role in maintaining the undifferentiated state of progenitor cells [4].

Development of the metanephros, i.e., the permanent kidney, starts in the mouse embryo at E10.5 when the nephric, or Wolffian duct, which arises from the intermediate mesoderm in the cranium region, starts budding towards the metanephric mesenchyme. The invading outgrowth of the nephric duct, the ureteric bud (UB), then reciprocally interacts with the surrounding mesenchymal and stromal cells and branches into a tree-like structure [5]. In the mature kidney, the hierarchically branched ureteric bud will function as the collecting duct system, while mesenchymal and stromal cells will differentiate to form the nephron. The nephron, the functional unit of the kidney, can be divided into glomerulus, proximal tubules, Loop of Henle, and distal tubular structure, via which it connects to the collecting duct. Vascularization and enervation of the developing mouse metanephros begin at E12.5 to E13.5. Measuring the tissue oxygen concentration in the embryo tissue is experimentally challenging and, therefore, not exactly known. Assuming the lack of vascularization and blood supply, one can expect that the early metanephros develops in a rather hypoxic environment [6].

Conventional tissue engineering approaches aiming to generate functional renal tissue have, up to this time, failed, largely due to the complexity of the kidney and the multiple cell types that constitute the adult kidney. Developmental engineering offers a strategy to facilitate the self-organized growth of functional tissue from progenitor cells, similar to organogenesis, utilizing findings from embryology in combination with tissue engineering [6,7,8]. The progress in regenerative medicine, especially in the fields of organoids and developmental engineering, brought focused attention to whole-organ culture.

In vitro culture of the kidney has been established in the middle of the last century as a method that allowed observational and experimental studies on a developing organ [9,10,11]. Using this technique helped to delineate renal organ development mechanisms and gave insight into the genes and endogenous factors involved [12]. Over time, the basic method of in vitro kidney culture, the Grobstein assay, has been modified for specific experiments, and culture parameters have been adjusted to optimize ex vivo development, tissue response, and growth of the organ [13,14]. Commonly in the Grobstein assay, the developing organ is cultured on a floating membrane covered by a thin layer of growth medium to maximize oxygen exposure. However, looking at the oxygen levels of in vitro cultured and in vivo grown embryonic kidneys, we hypothesized that high oxygen levels might impair the full potential of embryonic development. To perfect the self-organized growth of the embryonic metanephros during the early phase of development, it might be necessary to support vascularization in vitro and optimize oxygen concentrations to mimic the tissue situation in the embryo. The goal of our study was to analyze the influence of hypoxia on the early branching pattern of the developing mouse metanephros. Further, we tried to find the optimal time point and length of hypoxia culture of in vitro- grown kidneys to improve the branching patterns of the ureteric bud, and the growth and viability of the kidney progenitor subpopulations.

## 2. Materials and Methods

### 2.1. Animal Experiments

Animals were housed in the Veterinary Medical Unit at the Sepulveda Campus of the Greater Los Angeles Veterans Administration according to standardized specific pathogen-free conditions. Procedures involving animals were reviewed and approved by the Institutional Animal Care and Use Committee of the Greater Los Angeles Veterans Administration (Protocol #10121669). Researchers handling animals were trained and certified according to the guidelines of the Association for Assessment and Accreditation of Laboratory Animal Care (AALAC).

### 2.2. Isolation of Embryonic Kidneys

E11.5-13.5 embryonic kidneys were isolated from timed pregnancies of Tg (Hoxb7-Venus*)17Cos/J Mouse transgenic mice, which express the myristoylated yellow fluorescent protein, myr-Venus under control of the homeobox B7 promoter/enhancer. All animals were obtained from Jackson Laboratory (Bar Harbor, ME, USA). Embryonic kidneys were dissected and washed in cold (4 °C) 1× phosphate-buffered solution (PBS). Kidneys were prepared for the whole organ culture. One kidney of the kidney pair was cultured in a hypoxic chamber (Billups-Rothenberg, Modular Incubator Chamber) and exposed to hypoxic condition (5% O_2_, 5% CO_2_, 37 °C) and the other kidney was in a conventional incubator and exposed to normal condition (20% O_2_, 5% CO_2_, 37 °C). In brief, kidneys were transferred on 0.4 μm HTTP Isopore Membrane Filters (Merck Millipore Ltd., Tullagreen, Cork, Ireland) and cultured as float culture in high-glucose DMEM/F12 (Gibco, Invitrogen, Carlsbad, CA, USA) with 10% fetal bovine serum (FBS; Sigma-Aldrich, St. Louis, MO, USA) and penicillin–streptomycin (Pen/Strep; Sigma-Aldrich) in 24-well flat-bottom non-pyrogenic cell culture plates (Costar, Corning Ltd., Corning, NY, USA). Cell culture medium was replaced after 48–72 h.

### 2.3. Confocal Microscopy and Analysis of Branching Characteristics

Laser confocal fluorescence microscopy and 3D-stack imaging were performed using an Olympus FV1000 microscope (Olympus Life Science Solutions, Center Valley, PA, USA) in combination with Olympus Fv10-ASW 0.4 imaging software. Embryonic kidneys were made transparent using FocusClear™ clearing reagent (Cedarlane, Burlington, NC, USA). Images (1024 × 1024 pixels) were generated using a 10× objective (NA 1.4) (Olympus) and a sampling speed of 40.00 μs/pixel. Laser excitation wavelength 488 nm, scanning filter eGFP was used to detect Hoxb7Venus positive cell signal. 3D-image stacks were transformed to isotropic images using Fiji ImageJ 1.47n (Wayne Rasband, National Institute of Health, New York, NY, USA). Images were then subjected to branching analysis using gradient-vector-based software (TreeSurveyor) [15]. For details on process flow, image processing, and data extraction, see supplemental data (Supplementary Figures S1 and S2). Cell culture images have been taken with a Meiji TC5100 microscope (Meiji Techno, San Jose, CA, USA) with Moticam 5.0 and with Motic Images 2.0 software (Microscope World, Carlsbad, CA, USA).

### 2.4. Statistical Analysis

Each experiment has been realized 3 independent times, with n technical replicates each, where n is ≥3, depending on the experiments (the exact number is written in the figures).

For the real-time PCR for HIF1α, each biological replicate was measured in three technical replicates. ∆∆Ct was expressed as a x-fold change in Figure 5. SE was calculated as an estimate of the standard error of a log2 fold-change.

Data are presented as mean ± standard deviation (SD) or mean ± standard error (SE), as expressed in the figure captions. Comparisons between three or two groups were carried out using the Kruskall-Wallis test, or the Wilcoxon rank-sum test. *p* < 0.05 was considered statistically significant.

### 2.5. RNA Extraction and Gene Expression Analysis

Total RNA from embryonic kidneys was isolated using the TRIzol-method (Invitrogen Corp., Carlsbad, CA, USA). Embryonic kidney tissue was shock frozen in liquid nitrogen and homogenized in Trizol in a 0.5 mL Eppendorf tube. cDNA was generated using the High-Capacity cDNA Reverse Transcription Kit (Applied Biosystems Inc., Foster City, CA, USA) according to the manufacturer’s recommendation. RT-PCR was performed using iQ™-Sybr Green Supermix (Bio-Rad, Hercules, CA, USA) and iCycler (Bio-Rad). For details on RT-primers see Supplemental Information Table S1. GADPH or β-actin were used as reference genes to calculate ΔCt [16]. Gene expression results were hierarchically clustered using the gene expression function of Heatmapper [17] in combination with the distance-measure method according to Pearson to generate a red/green heatmap (Figure 6).

### 2.6. Hoxb7 Assay

Cell numbers of the metanephros-HoxB7 subpopulation were characterized by FACS analysis. Kidneys were disassociated and fixed in 4% paraformaldehyde (Sigma-Aldrich). FACS analysis was performed on a BD FACSJazz cell sorter (BD Biosciences, Franklin Lakes, NJ, USA) to measure the number of fluorescent cells (Hoxb7Venus).

## 3. Results

### 3.1. UB Branching Morphogenesis

**Figure 1:**
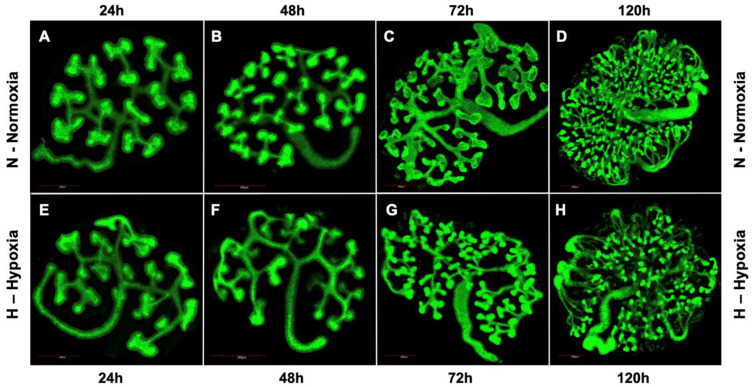
Kidney growth in normoxia and hypoxia. Embryonic kidneys exposed to hypoxic conditions during in vitro culture (**E**–**H**) exhibit different growth pattern compared to in vitro culture under normal conditions (**A**–**D**). Kidneys in hypoxic condition show increased branching from 24 h (**E**), but with a reduced overall and lower tip volume. Organs at 48, 72 and 120 h (**F**–**H**) present with stunted growth, reduced total volume and thinner collecting duct branches.

Confocal images of embryonic metanephros isolated at E12.5 and cultured in hypoxic conditions for 120 h appeared to have an altered development compared to metanephric from normal conditions (Figure 1). Metanephroi, after 24 h in hypoxia culture, show an increased length of the UB branches but appear to have a lower overall volume and exhibit reduced tip volume. At 48 h, the lengthening of the UB branches is more obvious, and a reduction in tip number becomes visible. At 72 h and 120 h, kidneys in hypoxia present stunted growth, lower overall volume, and thinner, more fragile-looking branches. The number of UB branching tips in hypoxic conditions is also visibly reduced compared to normal condition (Figure 1).

### 3.2. Calculated Branching Morphogenesis

**Figure 2:**
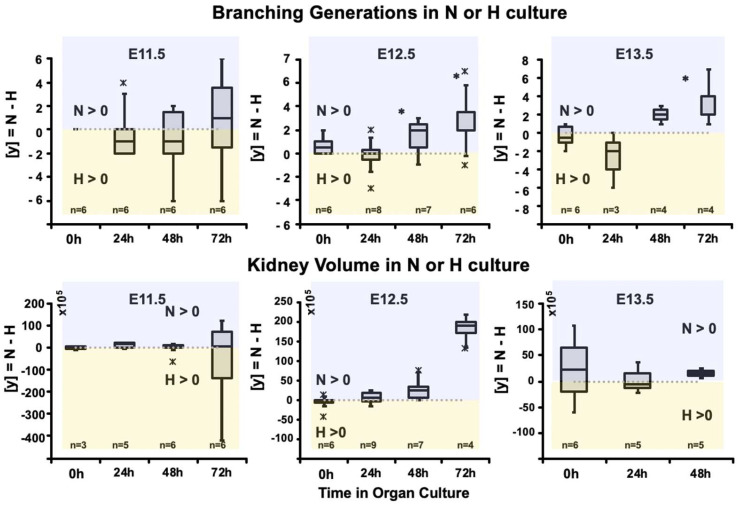
Branching generation and kidney volume. The upper panel shows the number of branching generations of embryonic kidneys harvested at E11.5, E12.5, and E13.5 after culture in normal (N) and hypoxic (H) condition. Branching generations are expressed in box plots as [y] = N−H at time points 0 h (fresh), 24 h, 48 h and 72 h. *: *p* < 0.05 Kidney volume (*y*-axis) is expressed as voxel.

We compared the branching generations and organ volume of embryonic kidneys isolated at E11.5, E12.5, and E13.5 using a gradient-vector-based software TreeSurveyor [15] and found that in kidneys isolated at E12.5, branching increased during the first 24 h and then significantly accelerated in normoxia (Figure 2). At 72 h, the number of branching generations was more significant in normal conditions than under hypoxia. No effect was observed on kidney volume at E11.5 and E13.5, while at E12.5, hypoxia seemed to decrease kidney volume. When kidneys from E13.5 are cultured for 72 h, they are too complex to be analyzed and therefore have not been included.

**Figure 3:**
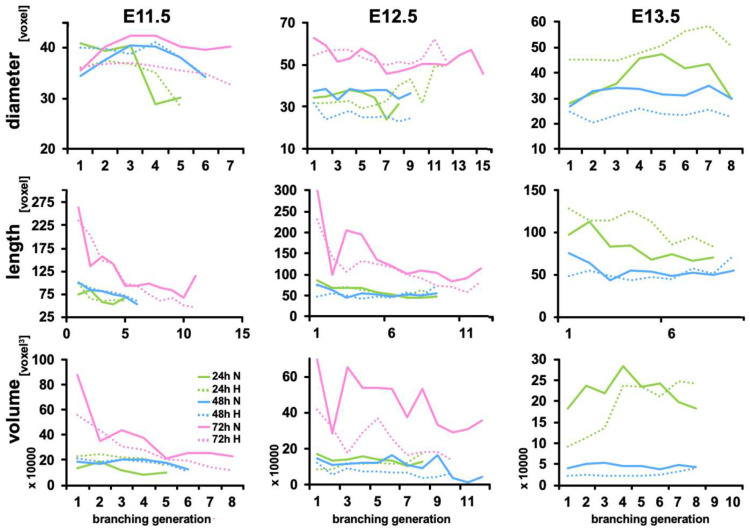
Diameter, length, and volume of ureteric bud branches. Analysis and comparison of ureteric bud branch diameter, length, and volume of kidneys E11.5, E12.5, and E13.5 after 24 h, 48 h, and 72 h of in vitro culture in normal or hypoxic conditions.

We also compared the volume, length, and diameter of individual branches of each generation of kidneys in hypoxia and normal conditions. Figure 3 shows that hypoxia had only a slight influence on the length and diameter of E11.5 and E12.5 UB branches. In E11.5 kidney, UB branches of kidneys cultured in hypoxia and normoxia have similar diameter and length after 24 and 48 h of culture. After 72 h of culture, the UB branches of kidneys in hypoxia have similar lengths than those in normoxia, but smaller diameters. 

In E12.5 kidneys, the diameter of the UB branches of 1st to 6th generations is smaller for kidneys cultured in hypoxia, while the diameters of the branches of 7th and 8th generations are bigger. After 48 h of culture, all branches of hypoxic kidneys have smaller diameters than their normoxic counterparts, but they will catch up, and after 72 h diameters become similar. We did not observe significant differences in the length of the branches between hypoxic and normoxic conditions.

In addition, branch lengths were constant in hypoxia and normal conditions during the first 24 h and 48 h. At 72 h, the branch lengths appear to decrease with the branching generations (Figure 3). In E13.5 kidneys, the branch dimensions (diameter and length) are bigger in hypoxic kidneys after 24 h of culture. However, after 48 h, the branch diameters have shrunk and are smaller in hypoxia. The volume of the branches appears to be similar in kidneys cultured under hypoxic and normal conditions, except for E12.5 kidneys after 72 h under hypoxic conditions that have smaller diameters than their normoxic counterparts.

### 3.3. Expansion of hoxb7+ Cells in Hypoxia

**Figure 4:**
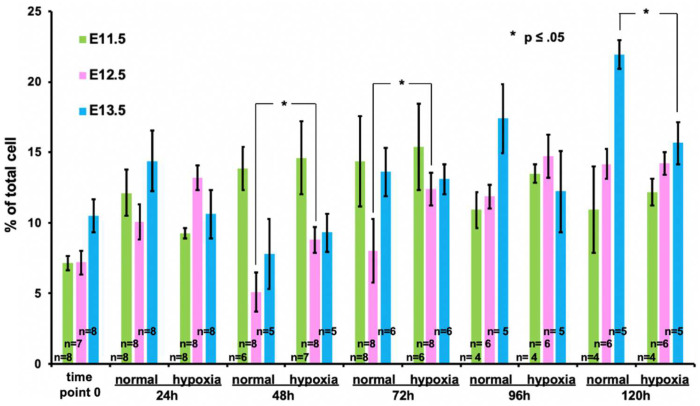
Percentage of Hoxb7+ kidney cells in normoxia vs. hypoxia. Hypoxic conditions exert influence on the growth of hoxb7+ cells in embryonic kidneys in in vitro culture. During the early stages of in vitro culture, hypoxia seems to increase the hoxb7+ cell population in the kidneys (time points 24 h and 48 h). At later time points, 96 h and 120 h, hypoxia seems to have the opposite effect, and hoxb7+ cells represent a smaller portion of the overall cell composition. In addition, hypoxia seems to affect kidneys isolated at E12.5 and E13.5 stronger than kidneys isolated at E11.5. Error bars represent SE.

The ureteric bud cells are a driver of renal development [5]. Their branching helps to establish the tree-like structure of the collecting duct and shapes the kidney from the renal pelvis outwards. To study the effect of hypoxia on the ureteric bud cell population of the developing kidney, we isolated kidneys from developing stages E11.5, E12.5, and E13.5 and cultured them under normal and hypoxic conditions for up to 120 h. Measuring the ureteric bud marker hoxb7, we compared the percentage of ureteric bud cells of kidneys from E11.5, E12.5, and E13.5 at different time points. We found that hypoxic conditions influence the growth of hoxb7+ cells during in vitro cultures of E12.5 and E13.5 kidneys but not E11.5 ones. (Figure 4). At early time points, 24 h and 48 h (and 72 h for E12.5 kidney), we found that hypoxia stimulates an increase in hoxb7+ cells. Longer hypoxia exposure of the embryonic kidneys seems to have a stunting effect on hoxb7+ cells. Hypoxia at 96 h and 120 h is correlated with a reduced hoxb7+ cell number. Kidneys from E12.5 and E13.5 seem to be more strongly affected by hypoxia than kidneys from E11.5, as hoxb7+ cell numbers from E11.5 under hypoxia do not differ much from normal conditions in later time points.

### 3.4. Induction of HIF1α

**Figure 5:**
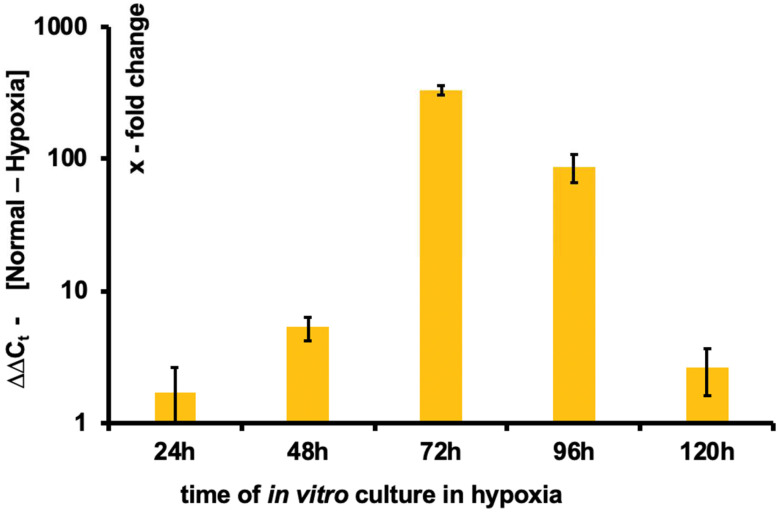
HIF1α mRNA in response to hypoxia. Quantitative analysis of hypoxia-inducible factor 1 subunit alpha (HIF1α) by real-time polymerase chain reaction (qRT-PCR) shows a gradual up-regulation of HIF1α mRNA in response to hypoxia. ∆Ct is the difference in amplification cycles (Ct) between the gene of interest and the endogenous control (β-actin) of a sample. ∆∆Ct refers to the Ct difference in an experimental sample (hypoxia) and the same gene in the control condition (normoxia). Error bars represent an estimate of the SE of a log2 fold-change.

Hypoxia-inducible factors (HIF) constitute a group of basic helix-loop-helix proteins that are oxygen-sensitive and have an important role in the transcriptional response to hypoxia [18]. Using quantitative real-time polymerase chain reaction (qRT-PCR) to compare the gene expression of hypoxia-inducible factor 1 subunit alpha (HIF1α) in the E12.5 kidneys under hypoxia and normal conditions, we found a strong up-regulation of HIF1α during the first 72 h in hypoxia. HIF1α was expressed 1.7-fold at 24 h, 5.3-fold at 48 h, and >300-fold at 72 h, after which expression slowly normalized (Figure 5).

### 3.5. Differentiation Markers

**Figure 6:**
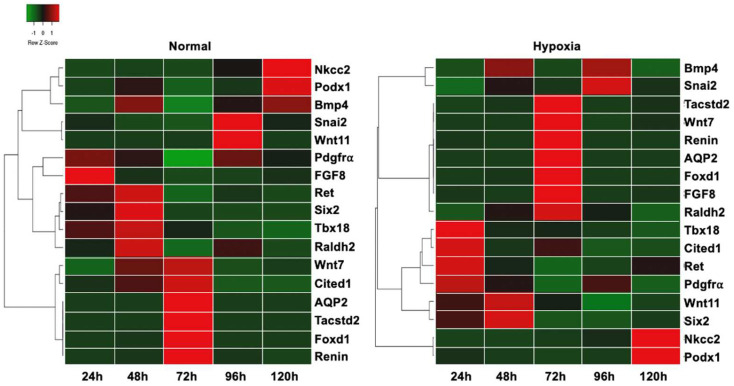
Gene expression of progenitor and differentiation markers of embryonic kidneys. Heatmap showing hierarchically clustered gene expression data of progenitor and differentiation markers of embryonic kidneys in hypoxic culture, compared to normal condition. Gene expression is presented for 24 h, 48, 72 h, 96 h, and 120 h. Genes that show higher expression in hypoxic condition than in normal control are in red, green is associated with a reduction in mRNA level.

To better understand the influence of hypoxia on the metanephros development and the differentiation of the renal progenitor subpopulations, we compared the expression levels of developmental markers of the kidney under normal conditions and hypoxia. Hierarchical cluster analysis and Pearson distance measure showed that culturing embryonic kidneys under hypoxia conditions seems to affect the gene expression of progenitor and differentiation markers of the ureteric bud, the metanephric mesenchyme, and the stromal cells (Figure 6).

**Figure 7:**
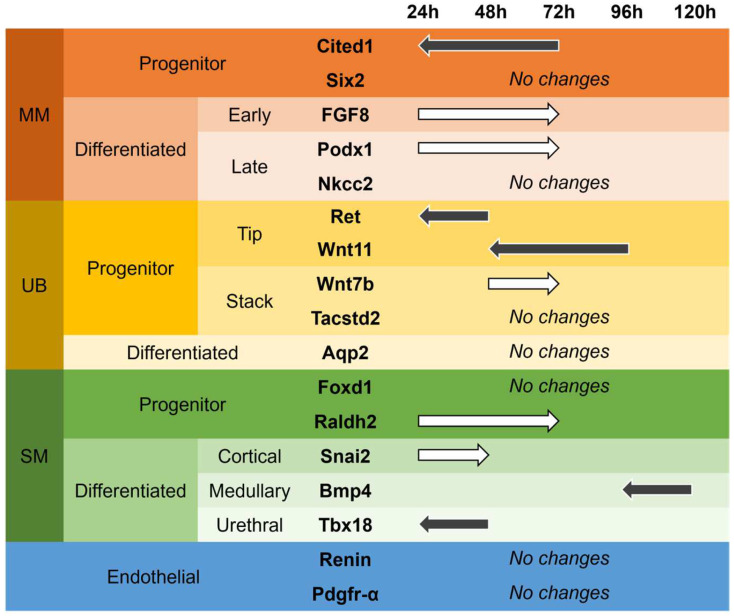
Chart of the expressional shift of developmental and the differentiation marker genes of the renal progenitor subpopulations in hypoxia vs. normal. Information is based on the qRT-PCR analysis above. Hypoxia causes UB tip branching markers Ret and Wnt11 to be expressed earlier, while a UB stalk marker Wntb7 is found delayed. MM differentiation marker FGF8 and Podx1 are delayed. Stroma progenitor cell marker Raldh2 is expressed later and the expression of stroma cell differentiation marker Snai2 is delayed, while stroma medullary marker Bmp4 and urethra marker Tbx18 are expressed earlier. Black arrow indicates an earlier gene expression in hypoxia compared to normoxia. White arrow indicates a later gene expression in hypoxia compared to normoxia.

In brief, the data (Figure 6) demonstrate that hypoxic culture condition up-regulates ureteric bud progenitor tip markers earlier than in normal conditions, as expression of signaling molecule Wnt11 and receptor tyrosine kinase c-Ret, both markers of the tip cells of the advancing ureteric bud, shifted from 96 h to 48 h, and from 48 h to 24 h, respectively (Figure 7). Expression of Wnt7b, a marker for the stalk aspect of the branching ureteric bud, was found delayed in hypoxic conditions, with the up-regulation shifting from 48 h to 72 h. However, the expression of the stalk marker Tumor-Associated Calcium Signal Transducer 2 (Tacstd2) was unchanged in hypoxia, similar to the gene expression of UB differentiation marker AQP2.

In the metanephric mesenchymal cells population, expression of the MM early differentiation marker Fibroblast Growth Factor 8 (FGF8) was delayed and found up-regulated at 72 h in hypoxia, instead at 24 h under normal conditions [19]. Late MM differentiation marker Podx1 was also delayed (from 24 h to 72 h) but Nkcc2 remained unchanged. Cited1, an MM progenitor marker that is observed when the ureteric bud invades the metanephric mesenchyme [20] is expressed earlier in hypoxia than under normal culture conditions. Interestingly, in normal conditions, Cited1 is slightly up-regulated at 48 h and strongly up-regulated at 72 h. Under hypoxic conditions, Cited1 is strongly up-regulated at t = 24 h and slightly up-regulated at 72 h. Additionally, Six2, which functions in maintaining the mesenchymal progenitor state, was unchanged in hypoxic culture [21].

In the stroma cell population, some markers also exhibited a different response in hypoxia when compared to normoxia. Under normal conditions, stromal cell progenitor marker Raldh2 shows up-regulated gene expression at 48 h of culture, while under hypoxic conditions, the up-regulation was observed at 72 h. However, Foxd1, another stroma cell progenitor marker, was unchanged. The expression of the cortical stroma marker snail family transcriptional repressor 2 (Snai2) was delayed, shifting from 24 h to 48 h. The medullary and urethral stroma cell differentiation markers Bone morphogenic protein 4 (Bmp4) and T-box transcription factor 18 (Tbx18), respectively, were observed to be expressed earlier; Bmp4 showed up-regulation at 96 h instead of 120 h, and urethral transcription factor Tbx18 was expressed after 24 h of hypoxic cultured, compared to 48 h under in normal conditions. Interestingly, despite changes in the gene expression of renal cell markers, no changes in the expression of endothelial cell markers, namely platelet-derived growth factor receptor-alpha (Pdgfr-⍺) and renin, were observed. Overall, out of seventeen quantified progenitor and differentiation markers, five were expressed earlier (Cited1, Ret, Wnt11, Bmp4, and Tbx18), five were expressed later (FGF8, Podx1, Wnt7b, Raldh2, and Snai2), and seven did not appear to be influenced by hypoxia (Six2, Nkcc2, Tacstd2, Aqp2, Foxd1, Renin, and Pdgfr-⍺) (Figure 7). For a different, maybe better visually understandably, view of the shifts and changes of marker cell expression, see Supplemental Figure S3.

## 4. Discussion

In mice, nephrogenesis is initiated with a branching phase between E11.5 to E15.5, during which most of the branching occurs. After E15.5, the branching rate slows down, and trunks stretch extensively; this is the collecting duct elongation phase [22]. Parts of mouse renal development can be recapitulated in vitro, commonly under standard conditions of 37 °C, 20% oxygen and 5% carbon dioxide. This model development, however, is making assumptions about the oxygen level in the developing tissue that is not supported by measurements of oxygen availability in the developing embryo, where 20% is the upper oxygen level and typical for the upper airway [1]. In contrast, the oxygen level in the cortical medullary region of the kidney is only at 1% [23]. In this study, we investigated the impact of long-term hypoxia on in vitro cultured embryonic kidneys.

In our study, Images gathered from embryonic metanephros isolated at E12.5 cultured in hypoxic conditions demonstrate visible changes to renal development compared to metanephroi cultured under normal conditions (Figure 1). We observed an increase in the length of the UB branches, which correlates with our finding from gene expression profiling that UB branching starts earlier and that MM differentiation is delayed. In the developing kidney, the generation of the renal architecture and the differentiation of nephrons from the metanephric mesenchyme is generated by the mutual induction of UB and MM [24]. As it can be seen in our study, under hypoxic conditions, the extended UB branching is prominent at 48 h, and we then observe a reduction in UB tip number. We suggest that this can be explained by a delay in the MM differentiation, as observed in a delay of MM differentiation marker FGF8 (Figure 7). The absence of MM differentiation signaling towards the UB progenitors results in extensive growth of the UB branches (Figure 1).

Based on those results, we suppose that hypoxia impacts kidney branching, but that it exerts little influence on the elongation phase. To test this hypothesis, we measured the level of mRNA expression of HIF1α, an oxygen-sensitive transcription factor that has a major role in the response to tissue hypoxia and is indispensable for normal embryo development, especially in the cardiovascular system [25,26]. Interestingly, we observed that HIF1α mRNA expression increases up to 500-fold at 72 h (i.e., E15.5, for kidney collected at E12.5, which corresponds to the branching phase) in hypoxia compared to normal conditions (Figure 5). In addition, we noticed that this expression decreases after 72 h (which corresponds to the elongation phase), supporting our hypothesis (Figure 5).

HIF1α has been shown to influence branching in the placenta [27]. It further has been demonstrated that HIF1α has different effects on kidney branching during development. Kurtzeborn et al. demonstrated that HIF1α increases the UB branching [28]. However, on the other hand, its overexpression impairs nephrogenesis [29]. Our results suggest that nephrogenesis impairment occurs mainly during the late branching phase. Indeed, at 24 h, E12.5 kidneys cultured in hypoxia generate more branches than kidneys in normoxia, but with smaller diameters. This finding is supported by a study on human embryonic renal tissue by Bernhardt et al., who demonstrate that expression of HIF1α was located to the tip of the branching UB branching structures [30]. After 48 h, though, hypoxia has a negative effect on the generation of UB branches, compared to kidneys in normoxic conditions, as kidneys in normal atmosphere have more branching and are bigger than their hypoxic counterparts. After 72 h, we observed a normalization of HIF1α mRNA expression, which can be explained by the increase of the volume of the kidneys in normal conditions, vs. the smaller size of the hypoxic kidneys. This increase in volume in normal kidneys potentially increases hypoxia in the center of the tissue which in turn would increase the expression of HIF1α mRNA expression. This explanation is supported by the finding, that HIF1α expression is low in the outer cortex and stronger in the medullary tubular compartment and the collecting duct [18].

When we compared gene expression levels of kidneys in hypoxia with normoxia, we observed that most differences occur during the first 72 h, supporting our initial imaging observations. In the UB cell population, gene expression of tip UB cells was found upregulated in the early time of in vitro culture, while the expression of Wnt7 mRNA, a UB stalk marker, was delayed to 72 h, suggesting a higher UB branching activity during the first 24 h of culture that lasts less time, which corroborates the branching analysis. Signaling molecule Wnt11 and receptor tyrosine kinase c-Ret are markers of the tip cells of the advancing ureteric bud. In hypoxic culture conditions, c-Ret expression shifted from 48 h to 24 h, and the expression of Wnt11 was up-regulated after 48 h instead of after 96 h under normal conditions. This suggests that hypoxia up-regulates UB progenitor tip cells, which signal the MM cells to maintain undifferentiated status [31], delaying their differentiation. In return, undifferentiated MM cells positively regulate the self-renewal of the UB tip cells [32], enhancing the UB branching. After 48 h, hypoxia has a negative effect on nephrogenesis. We observed fewer UB tip markers associated with less branching and smaller kidneys in hypoxia than in normal conditions. We believe that the overexpression of HIF1α upregulated proteins from the Spry family [33], well-known negative regulators of receptor tyrosine kinase-mediated signals [33], such as Ret receptor of GDNF, which has been correlated with a diminished number of glomeruli and reduced kidney size [34].

Wnt7b functions as a signaling molecule that stabilizes the cortico-medullary axis in the kidney. It is expressed in the epithelial cells of the collecting duct. In hypoxic conditions, the expression of Wnt7b appeared delayed and was shifted back from 48 h to 72 h, suggesting a slowing of the medulla development and loop of Henle development, as it has been shown in knock-out studies [35]. Due to the role of Wnt7b in the activation of the canonical Wnt signaling pathway, one can speculate that hypoxia causes a delay in the formation of the medullary zone of the kidney. A second UB stalk marker, (Tacstd2), and the collecting duct water channel protein (AQP 2), a differentiation marker, were unchanged (Figure 6 and Figure 7, and Table S2).

In addition, we observed an early up-regulation of the MM progenitor cell marker Cited1, a member of the cAMP Response Element-Binding Protein (CREB) family of transcription factors. Expression of Cited1 is downregulated when the nephron progenitor cells differentiate towards a nephron epithelial phenotype. In hypoxia, Cited 1 was found to be up-regulated earlier at 24 h, downregulated at 48 h, and activated again at 72 h, suggesting a wave of maintenance of progenitor status and epithelial differentiation throughout organ development (Figure 6 and Figure 7, and Table S2). However, the expression of Sine Oculis Homeobox Homolog 2 (SIX2), a progenitor marker of the MM cells that give rise to the cells of the nephron, remained unchanged. The early MM differentiation marker FGF8, a growth factor essential for cell survival and gene regulation [19], exhibited delayed expression in hypoxia. In the developing kidney, FGF8 expression starts early after UB invasion and marks the formation of S-shape bodies in the differentiating mesenchymal mesenchyme. In our experiment, the hypoxia condition seems to shift FGF8 expression to 72 h, suggesting a delay in nephron formation and an extension of the progenitor state of the MM cells. Podocalyxin-like protein 1 (Podx1) marks a later stage of MM differentiation and remained unchanged by hypoxia, similar to the Na-K-Cl-cotransporter protein 2 (Nkcc2), found in the ascending aspect of the loop of Henle. Taken together, the downregulation of MM progenitor cell markers and the delay of differentiation markers suggest that hypoxia delayed the differentiation of MM cells into the nephron. At 72 h, we observed fewer MM progenitor cells and more MM differentiated cells, as well as more UB stalk cells. The kidneys exhibited a reduction in branching and a smaller volume than their counterparts cultured in normal conditions. It is possible that the peak of the hypoxic factor stimulated stalk cells and MM differentiation through vascular endothelial growth factor A (VEGFA) and neurogenic locus notch homolog protein 1 (NOTCH1) [35]. Previous studies have shown that forced expression of ectopic NOTCH1 within MM cells is followed by precocious differentiation of the nephron progenitor cells (NPCs) [36,37], and that VEGFA and blood vessels signal to maintain truck epithelium in pancreatic development, an organ with a similar development to ureteric branching [38]. After 72 h, we observed very few differences. In vivo, this time point corresponds to the collecting duct elongation phase [22]. We conclude that hypoxia is impactful in the branching phase of kidney development but has a limited effect on the elongation phase.

Stroma progenitor markers Raldh2 appear also later in hypoxic conditions (72 h compared to 48 h in normal conditions). Together with Raldh3, Raldh2 is responsible for ventral retinoic acid generation in the caudal region of the embryo [39] and is important for regulating branching morphogenesis [40]. The expression of the cortical stroma marker Snai2, a zinc-finger transcription factor, that is crucial in epithelial-mesenchymal transition during organ formation, is delayed in normal conditions to 96 h, while it is periodically up- and downregulated every 24 h in hypoxia (Figure 6 and Figure 7 and Table S2). Bmp4 regulation switched and is up-regulated at 24 and 48 h, while it is up-regulated only at 96 and 120 h under normal conditions. Tbx18, which regulates the development of ureteral mesenchyme, is up-regulated earlier at 72 h. Interestingly, no effect was observed on the late stroma cell markers, which also have a role in the developing vascular system, like renin, the kidney-expressed transcription factor Foxd1, and Pdgfr-⍺. The expression of renin in the developing kidney is correlated with the formation of the renal vasculature and is observed at E14 in α-smooth muscle-producing cells arteries [41]. Pdgfr-⍺ has an important role in the development of the glomerular and tubulointerstitial compartments, where it recruits mesenchymal cells, and in the regulation of vascular permeability and cell migration [42]. Foxd1, an upstream regulator of the renin-angiotensin system, is essential during renal development [43], where Foxd1+ positive cells, known as pericytes, contribute to the capillary basement membrane [44]. The unchanged gene expression of renin, Pdgfr-⍺, and Foxd1 suggests that in our model, hypoxia has no effect on the formation of the renal vascular system.

## 5. Conclusions

In this study, we addressed the possibility that adjusting the oxygen supplied to embryonic kidneys cultured in vitro could improve growing conditions. We concluded that reducing the culture oxygen concentration to mimic the in vivo environment has varying effects on the different subpopulations of embryonic kidney cells at specific developmental stages.

We also concluded that although standard conditions are not optimal for the in vitro culture of renal explants, they do give a good average model for development. However, we believe that implementing hypoxic conditions can positively influence kidney in vitro maturation, as observed in other tissues [45] or cell culture [3] and further experiments are ongoing to confirm this hypothesis and to further characterize the effects of hypoxia on different subpopulations of cells during kidney development. However, our results suggest that organ culture oxygen concentrations need to be adjusted to physiological conditions to enable optimal growth, and thus, may have to be changed during the course of the culture, as observed for other tissues [46,47]. However, additional detailed studies are necessary to delineate the use of gradual changes in oxygen conditions on the branching process and nephron development.

## Data Availability

Not applicable.

## Supplementary Materials

The following supporting information can be downloaded at: https://www.mdpi.com/article/10.3390/bioengineering9120801/s1, Figure S1: Flow Chart Branching Analysis; Figure S2: Branching Analysis; Table S1: Reverse Transcription Primers; Table S2: Roles of Differentiation Markers; Figure S3: Shift in Gene Expression.
