# Supplementary material for: Effect of Hypoxia on Branching Characteristics and Cell Subpopulations during Kidney Organ Culture
Source: Bioengineering (Basel). 2022 Dec 14;9(12):801. doi: 10.3390/bioengineering9120801 (PMC9774677; doi:10.3390/bioengineering9120801)
Supplement: Supplementary file 1 [file bioengineering-09-00801-s001.zip › bioengineering-2006232-supplementary.pdf]

bioengineering

Article

Effect of Hypoxia on Branching Characteristics and Cell Subpopulations during Kidney Organ Culture

Morgan Hamon [1,2,*], Hsiao-Min Cheng [1,2], Ming Johnson [1], Norimoto Yanagawa [1,2], Peter V. Hauser [1,2,*]

[1] Medical and Research Services, Greater Los Angeles Veterans Affairs Healthcare System at Sepulveda, North Hills, CA, 91344, USA
[2] Department of Medicine, David Geffen School of Medicine, University of California Los Angeles, Los Angeles, CA, 90095, USA
* Correspondence: morganhamon@ucla.edu (M.H.); pvhauser@ucla.edu (P.V.H.)

Supplementary

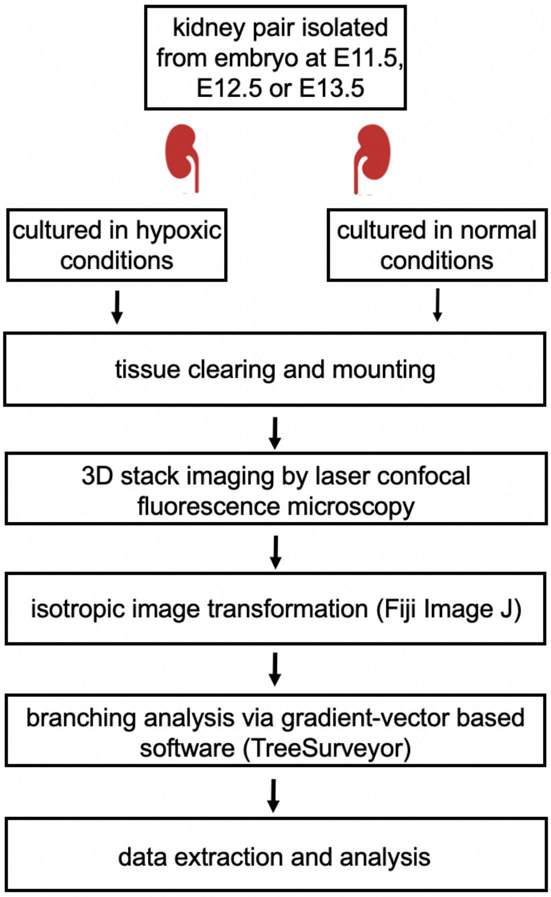

Figure S1. Flow Chart Branching Analysis. Flow chart of the experimental procedure for the branching analysis. Kidney pairs were isolated from time pregnancies at E11.5, E12.5 and E13.5. One kidney was cultured under hypoxic and one in regular conditions. After in vitro culture, the kidneys were fixed, cleared and mounted. 3D stack imaging was performed by laser confocal microscopy. Image were transformed into isotropy using Fiji Image J. Subsequent analysis was performed using TreeSurveyor.

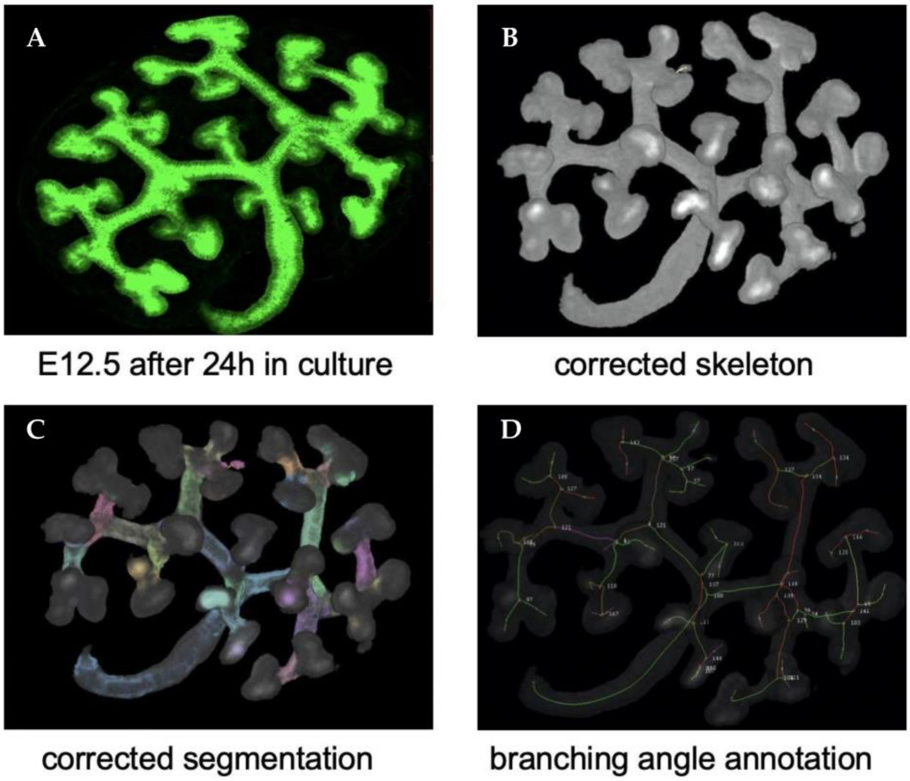

Figure S2. Branching Analysis. A Z-stack image of the embryonic kidney is generated by confocal microscopy (**A**) and the layers are transformed to isotropic images. The transformed isotropic Z-stack is then analyzed using gradient vector-based software (TreeSurveyor) and preliminary hierarchical branched structure is generated [15]. Errors can be edited and a corrected skeleton (**B**) is used to extract segmentation (**C**), branching length, branching angle (**D**) and other parameters from the 3D image.

Table S1. Reverse Transcription Primers.

Target	Forward	Reverse
β-actin	TTGCTGACAGGATGCAGA	ATCTGCTGGAAGGTGGACAG
AQP 2	GCCACCTCCTTGGGATCTAT	TGTAGAGGAGGGAACCGATG
Bmp4	TGAGAGACCCCAGCCTAAGAC	CGAATGGCACTACGGAATGG
Cited 1	TATGTCGAGGCCTGCACTTG	AAGGTTGGAGTAGGCCAGAG
FoxD1	TTCGGATTCTTGGACCAGAC	CAAGTCAGGGTTGCAGCATA
GAPDH	TGAACGGATTTGGCCGTATTG	ACCATGTAGTTGAGGTCAATGAAG
HIF 1α	CCAGCAGACTCAAATACAAGAACC	TGTATGTGGGTAGGAGATGGAGAT
FGF8	TGGAAGCAGAGTCCGAGTTC	ATACGCAGTCCTTGCCTTTG
Nkcc2	GATGCAGAACTGGAAGCAGTC	GGCTCTGGAGTGTTCCTGTAAG
Pdgfrα	AAGACCTGGGCAAGAGGAAC	GAACCTGTCTCGATGGCACT
Podxl	TCCTTGTTGCTGCCCTCTAC	TTCCAAGGTTGGGTTGTCAT
Raldh2	AGATGCTGACTTGGACTACG	TCTGAGGACCCTGCTCAGTT
Ret	TTCTGAAGACAGGCCACAGGA	CACTGGCCTCTTGTCTGGCT
Renin	CTGGGCACTCTTGTTGCTCT	GGACCACGGGGGAGATAAA
Snai2	GCCTCCAAGAAGCCCAACTA	GCCGACGATGTCCATACAGT
Six 2	CAAGTCAGCAACTGGTTCAAGA	ACTGCCATTGAGCGAGGA
Tacstd2	ACTGTACATGCCCCACCAAC	GCAGGCACTTGGAAGTTAGC
Tbx18	GGATATTGTGCCGGTGGACA	CCGGGGAATCAGCATTTCCT
Wnt7b	TACCTAAGTTCCGCGAGGTG	AGGCTTCTGGTAGCTGCGTA
Wnt 11	ACATGCGCTGGAACTGCT	GCATACACGAAGGCTGACTC

Table S2. Roles of Differentiation Markers. Table recapitulating the embryonic kidney cell subpopulation markers' function.

Name	Full name	Function
**HIF1-α**	Hypoxia-inducible factor alpha	Oxygen-sensitive transcription factor
**Cited1**	Cbp/P300 Interacting Transactivator with Glu/Asp Rich Carboxy-Terminal Domain 1	Self-renewal of nephronic progenitor cells of the embryonic *kidney*
**Six2**	Sine Oculis Homeobox Homolog 2	Self-renewal of nephronic progenitor cells of the embryonic *kidney*
**FGF8**	Fibroblast Growth Factor 8	Coordination of the behavior of nephron progenitor cells during kidney development.
**Podx1**	Podocalyxin-like protein 1	Component of glomerular podocytes
**Nkcc2**	Na-K-Cl cotransporter	Aid in the secondary active transport of sodium, potassium, and chloride into cells
**c-Ret**	rearranged during transfection	Receptor tyrosine kinase
**Wnt11**	Wnt Family Member 11	Maintenance nephron progenitor niche integrity
**Wnt7b**	Wnt Family Member 7b	Development of the medullary component of the mouse kidney
**Tacstd2**	Tumor-Associated Calcium Signal Transducer 2	Unresolved physiological function
**AQP2**	Aquaporin 2	Reabsorbtion water from the urine
**Foxd1**	Forkhead Box D1	Promotion of nephron progenitor differentiation
**Raldh2**	retinaldehyde dehydrogenase 2	Catalyzation of the synthesis of retinoic acid (RA) from retinaldehyde
**Snai2**	Snail Family Transcriptional Repressor 2	Zinc finger transcription factor
**BMP4**	Bone morphogenetic protein 4	Transforming growth factor
**Tbx18**	T-Box Transcription Factor 18	Regulation of the development of the ureteral mesenchyme
**Renin**		Regulation of the body's mean arterial blood pressure
**Pdgfr-α**	Platelet-derived growth factor receptor-alpha	Cellular growth and differentiation

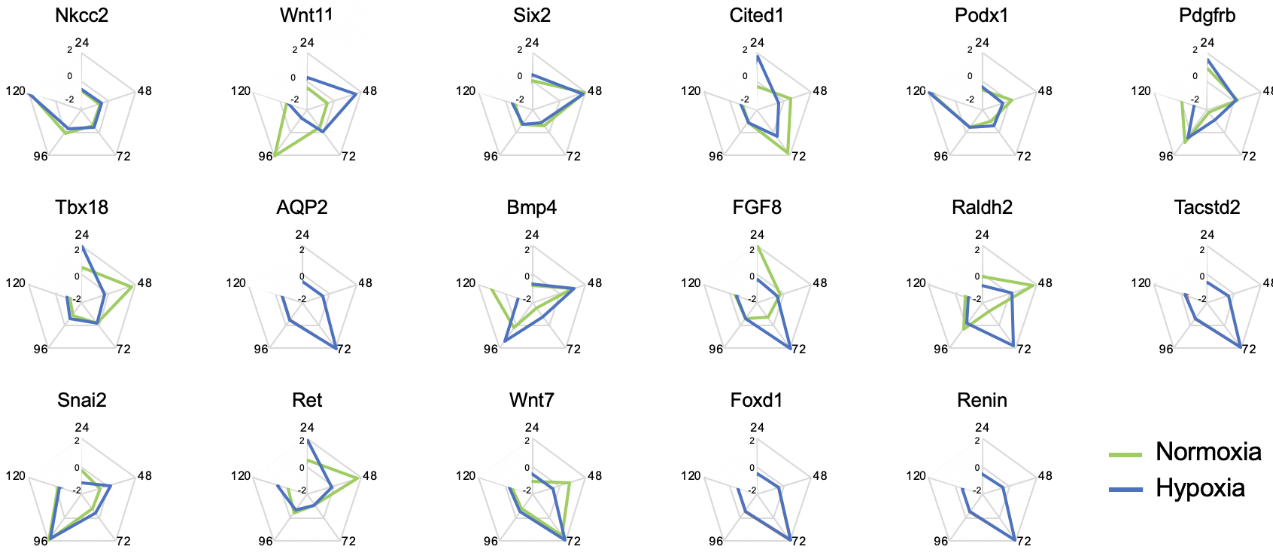

Figure S3. Shift in Gene Expression. Visualization of the shift in developmental and the differentiation marker gene expression via spider plots. Corners of the open pentagram represent a timepoint (24h, 48h, 72h, 96h and 120h). Normal gene expression levels are shown by a green line, a blue line shows the gene expression under hypoxic conditions.
